# Supplementary material for: A Chimeric ORF Fusion Phenotypic Reporter for Cryptococcus neoformans
Source: J Fungi (Basel). 2024 Aug 12;10(8):567. doi: 10.3390/jof10080567 (PMC11355724; doi:10.3390/jof10080567)
Supplement: Supplementary file 1 [file jof-10-00567-s001.zip › jof-3121800-supplementary.pdf]

Supplementary Materials

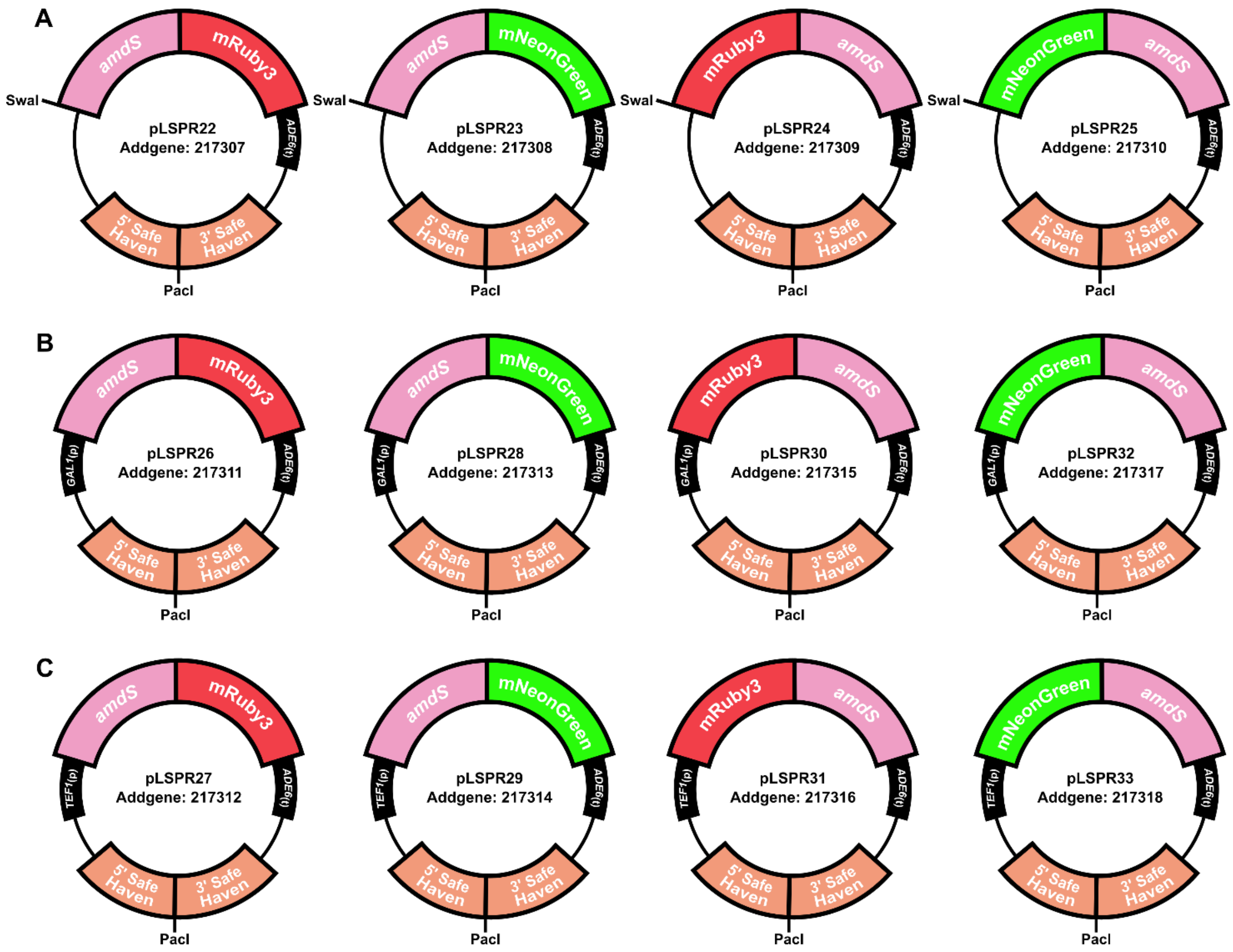

Supplementary Figure S1: Schematics of finalised plasmid maps for fusion reporter constructs. All listed constructs are available on Addgene database. (**A**) Promoter-less fusion reporter constructs for variant promoter combinations, both initial and flipped orientations. (**B**) Fusion reporters driven by *GAL7*$_{(p)}$ for both initial and flipped orientations. (**C**) Fusion reporters driven by *TEF1*$_{(p)}$ for both initial and flipped orientations.

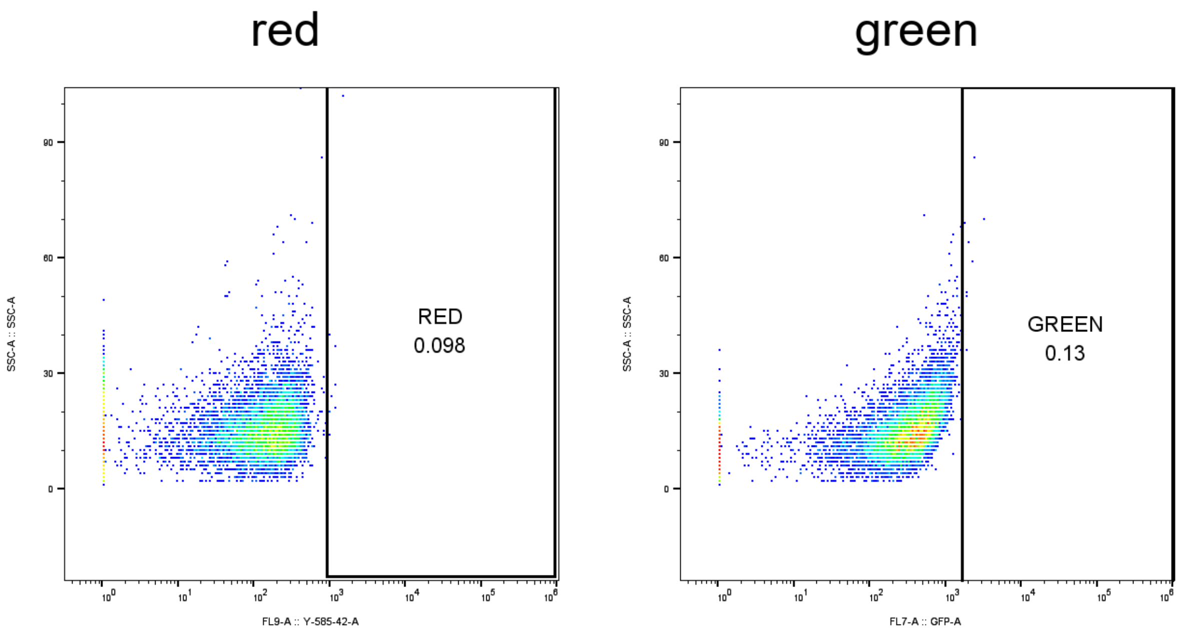

Supplementary Figure S2: Flow cytometry gating strategy employed for mRuby3 and mNeonGreen mutant *C. neoformans* cells. Population displayed is WT H99O 10th hour growth sample.

Supplementary Table S1: Plasmids used in this study.

Plasmid	Description	Backbone	Source
pSDMA57	Safe Haven homology vector with G418 resistance gene (*NEO*)	pSDMA25	(Arras *et al.*, 2015)
pLSPR22	no promoter + *amdS* + mRuby3 Reporter construct	pSDMA57	This study
pLSPR23	no promoter + *amdS* + mNeonGreen Reporter construct	pSDMA57	This study
pLSPR24	no promoter + mRuby3 + *amdS* Reporter construct	pSDMA57	This study
pLSPR25	no promoter + mNeonGreen + *amdS* Reporter construct	pSDMA57	This study
pLSPR26	*GAL7*$_{(p)}$ + *amdS* + mRuby3 Reporter construct	pSDMA57	This study
pLSPR27	*TEF1*$_{(p)}$ + *amdS* + mRuby3 Reporter construct	pSDMA57	This study
pLSPR28	*GAL7*$_{(p)}$ + *amdS* + mNeonGreen Reporter construct	pSDMA57	This study
pLSPR29	*TEF1*$_{(p)}$ + *amdS* + mNeonGreen Reporter construct	pSDMA57	This study
pLSPR30	*GAL7*$_{(p)}$ + mRuby3 + *amdS* Reporter construct	pSDMA57	This study
pLSPR31	*TEF1*$_{(p)}$ + mRuby3 + *amdS* Reporter construct	pSDMA57	This study
pLSPR32	*GAL7*$_{(p)}$ + mNeonGreen + *amdS* Reporter construct	pSDMA57	This study
pLSPR33	*TEF1*$_{(p)}$ + mNeonGreen + *amdS* Reporter construct	pSDMA57	This study

Supplementary Table S2: Strains used in this study.

Strain	Genotype	Source
H99O	Wild type *Cryptococcus neoformans MAT*α	(Janbon *et al.*, 2014)
LSPR16	*GAL7*$_{(p)}$ + *amdS* + mRuby3 in *C. neoformans* Safe Haven	This study
LSPR17	*TEF1*$_{(p)}$ + *amdS* + mRuby3 in *C. neoformans* Safe Haven	This study
LSPR18	*GAL7*$_{(p)}$ + *amdS* + mNeonGreen in *C. neoformans* Safe Haven	This study
LSPR19	*TEF1*$_{(p)}$ + *amdS* + mNeonGreen in *C. neoformans* Safe Haven	This study
LSPR20	*GAL7*$_{(p)}$ + mRuby3 + *amdS* in *C. neoformans* Safe Haven	This study
LSPR21	*TEF1*$_{(p)}$ + mRuby3 + *amdS* in *C. neoformans* Safe Haven	This study
LSPR22	*GAL7*$_{(p)}$ + mNeonGreen + *amdS* in *C. neoformans* Safe Haven	This study
LSPR23	*TEF1*$_{(p)}$ + mNeonGreen + *amdS* in *C. neoformans* Safe Haven	This study

Supplementary Table S3: Oligonucleotides used for this study.

Primer ID	Description	Sequence
UQ2962	Safe Haven forward primer	GGGTATGCCACAGATGCAGAT
UQ2963	Safe Haven reverse primer	TTGGATCCTCAATTGTCTCCT
UQ5391	SwaI cutsite 5' *amdS* forward primer	GGCCCCCCCTCGAATTTAAATATGCCTCAGTCCTGGGAGGAG
UQ5392	Linker + 5' mRuby3 forward primer	TCTGGCGGTGGTGGCTCCGGCATGGTCTCTAAGGGCGAGGAG
UQ5393	Linker + 3' *amdS* reverse primer	GGAGCCACCACCGCCAGAACCCTAAGGAGTGACGACGTTACC
UQ5394	*ADE6*(t) reverse primer	AACAAAAGCTGGAGCTCCACCGATGGGCTTGAAGACTAGGAA
UQ5395	Linker + 5' mNeonGreen forward primer	TCTGGCGGTGGTGGCTCCGGCATGGTCTCCAAGGGCGAGGAG
UQ5397	*TEF1*(p) forward primer	GGGTACCGGGCCCCCCCTCGACATTCTATGCTATACGGTACA
UQ5398	*amdS* + *TEF1*(p) reverse primer	CTCCTCCCAGGACTGAGGCATTTTGAAGTTTTCTGTGGAGAT
UQ5399	*GAL7*(p) forward primer	GGGTACCGGGCCCCCCCTCGATAGTGAAGTCGTATTGTCTCT
UQ5400	*amdS* + *GAL7*(p) reverse primer	CTCCTCCCAGGACTGAGGCATTCTCAGGAGAGAATTGAGTGC
UQ5992	SwaI cutsite + mNeonGreen forward primer	GGCCCCCCCTCGAATTTAAATATGGTCTCCAAGGGCGAGGAG
UQ5994	Linker + *amdS* forward primer	TCTGGCGGTGGTGGCTCCGGCATGCCTCAGTCCTGGGAGGAG
UQ5995	*amdS* + *ADE6*(t) overhang reverse primer	CACTATTACGCGCTCAACCTTCTAAGGAGTGACGACGTTACC
UQ5996	*amdS* overhang + *ADE6*(t) forward primer	GGTAACGTCGTCACTCCTTAGAAGGTTGAGCGCGTAATAGTG
UQ5997	mNeonGreen + *GAL7*(p) reverse primer	CTCCTCGCCCTTGGAGACCATTCTCAGGAGAGAATTGAGTGC

UQ5998	mNeonGreen + *TEF1*$_{(p)}$ reverse primer	CTCCTCGCCCTTGGAGACCATTTTGAAGTTTTCTGTGGAGAT
UQ6085	Linker + mRuby3 reverse primer	GGAGCCACCACCGCCAGAACCCTTGTAGAGCTCGTCCATGCC
UQ6086	mNeonGreen linker reverse primer	GGAGCCACCACCGCCAGAACCCTTGTAAAGCTCGTCCATGCC
UQ6087	Linker + *amdS* reverse primer	GGAGCCACCACCGCCAGAACCAGGAGTGACGACGTTACCGAG
UQ6088	SwaI cutsite + mRuby3 forward primer	GGCCCCCCCTCGAATTTAAATATGGTCTCTAAGGGCGAGGAG
UQ6097	mRuby3 + *GAL7*$_{(p)}$ reverse primer	CTCCTCGCCCTTAGAGACCATTCTCAGGAGAGAATTGAGTGC
UQ6098	mRuby3 + *TEF1*$_{(p)}$ reverse primer	CTCCTCGCCCTTAGAGACCATTTTGAAGTTTTCTGTGGAGAT

Supplementary Table S4: gBlock sequences used for this study.

gBlock ID	Description	Sequence
UQg48	mRuby3 crypto codon-optimised	GATCTCCACAGAAAACTTCAAAATGGTCTCTAAGGGCGAGG AGCTCATCAAGGAGAACATGCGTATGAAGGTCGTCATGGAG GGTTCTGTCAACGGCCACCAGTTCAAGTGCACTGGTGAGGG TGAGGGCAGGCCCTACGAGGGTGTCCAGACCATGAGGATCA AGGTCATCGAGGGTGGTCCCCTCCCTTTCGCCTTCGACATT CTTGCCACCTCTTTCATGTACGGCTCCCGTACCTTCATCAA GTACCCCGCCGACATCCCTGACTTCTTCAAGCAGTCCTTCC CTGAGGGTTTCACTTGGGAGAGGGTTACCAGGTACGAGGAC GGTGGTGTCGTCACCGTCACCCAGGACACCTCCCTTGAGGA CGGCGAGCTCGTCTACAACGTCAAGGTCAGGGGCGTTAACT TCCCCTCCAACGGTCCCGTCATGCAGAAGAAGACCAAGGGT TGGGAGCCTAACACTGAGATGATGTACCCTGCTGACGGTGG TCTCAGGGGTTACACTGACATCGCTCTCAAGGTTGACGGTG GTGGCCACCTCCACTGCAACTTCGTCACTACTTACAGGTCT AAGAAGACCGTCGGCAACATCAAGATGCCCGGTGTCCACGC CGTTGACCACCGTCTCGAGAGGATCGAGGAGTCCGACAACG AGACCTACGTTGTCCAGAGGGAGGTCGCTGTTGCCAAGTAC TCCAACCTTGGTGGTGGCATGGACGAGCTCTACAAGTAGAA GGTTGAGCGCGTAATAGTGG
UQg57	*amdS* codon-optimised with terminator	GATCTCCACAGAAAACTTCAAAATGCCTCAGTCCTGGGAGG AGCTCGCCGCTGACAAGCGTGCCCGTCTCGCTAAGACCATC CCTGACGAGTGGAAGGTCCAGACCCTCCCTGCCGAGGACTC CGTTATTGACTTCCCTAAGAAGTCTGGCATCCTTTCTGAGG CCGAGCTCAAGATCACTGAGGCCTCCGCTGCTGACCTTGTC TCCAAGCTCGCCGCCGGTGAGTTGACCTCTGTCGAGGTTAC CCTTGCTTTCTGTAAGCGAGCTGCTATCGCCCAGCAGTTGA CTAACTGCGCCCACGAGTTCTTCCCTGACGCCGCTCTCGCC CAGGCTAGGGAGCTCGACGAGTACTACGCTAAGCACAAGAG GCCCGTTGGTCCTCTCCACGGCCTCCCCATCTCTCTCAAGG ACCAGCTTCGAGTCAAGGGCTACGAGACTTCTATGGGCTAC ATCTCTTGGCTTAACAAGTACGACGAGGGCGACTCTGTTCT CACTACCATGCTCCGTAAGGCCGGTGCCGTCTTCTACGTCA

	AGACCTCTGTCCCCCAGACCCTCATGGTCTGCGAGACTGTC<br>AACAACATCATCGGCCGTACCGTCAACCCTCGTAACAAGAA<br>CTGGTCTTGCGGCGGCTCCTCTGGTGGTGAGGGTGCCATCG<br>TTGGCATTCGTGGTGGCGTCATCGGTGTTGGTACCGACATC<br>GGTGGCTCTATTCGAGTCCCCGCCGCCTTCAACTTCCTCTA<br>CGGTCTTAGGCCCTCCCACGGCCGACTCCCCTACGCTAAGA<br>TGGCCAACTCCATGGAGGGTCAGGAGACCGTCCACTCCGTT<br>GTCGGCCCCATTACCCACTCTGTTGAGGACCTCCGTCTCTT<br>CACCAAGTCCGTCCTCGGTCAGGAGCCTTGGAAGTACGACT<br>CCAAGGTCATCCCCATGCCCTGGCGTCAGTCCGAGTCTGAC<br>ATTATTGCCTCCAAGATCAAGAACGGCGGCCTCAACATCGG<br>CTACTACAACTTCGACGGCAACGTCCTTCCTCACCCTCCTA<br>TCCTCCGTGGCGTCGAGACCACCGTCGCCGCTCTCGCCAAG<br>GCCGGTCACACCGTCACCCCCTGGACCCCTTACAAGCACGA<br>CTTCGGCCACGACCTCATCTCCCACATCTACGCCGCTGACG<br>GCTCCGCCGACGTTATGCGTGACATCTCCGCTTCCGGCGAG<br>CCCGCCATTCCTAACATCAAGGACCTTCTCAACCCCAACAT<br>CAAGGCTGTTAACATGAACGAGCTCTGGGACACCCACCTCC<br>AGAAGTGGAACTACCAGATGGAGTACCTTGAGAAGTGGCGA<br>GAGGCTGAGGAGAAGGCCGGCAAGGAGGTGAGTAGATCACA<br>ATTTGTAACAGGTTGGATGATAAGTCCCCGGTCTAACATGT<br>AGCGACGACACTGAAGCTCACTTCTTGTAGCTCGACGCCAT<br>CATCGCCCCCATTACCCCTACCGCTGCCGTTCGACACGACC<br>AGTTCCGATACTACGGCTACGCCTCTGTCATCAACCTCCTC<br>GACTTCACCTCCGTCGTTGTTCCCGTTACCTTCGCCGACAA<br>GAACATCGACAAGAAGAACGAGTCCTTCAAGGCCGTTTCCG<br>AGCTTGACGCCCTCGTCCAGGAGGAGTACGACCCCGAGGCC<br>TACCACGGCGCTCCCGTTGCTGTCCAGGTTATCGGTCGAAG<br>GCTCTCCGAGGAGAGGACCTTGGCCATTGCTGAGGAGGTCG<br>GCAAGTTGCTCGGTAACGTCGTCACTCCTTAGGTCAAGTTG<br>TATTACATGCATCATAACTGTGTGACCTTGAATTGTGTCGA<br>GATCTCCTGCACAGATGAGCCCTGCAAAAGATGTTCATGTA<br>GCCATTTAATATAATGATAGACTTGGATCGGTCACGCGACG<br>TTGTTTTCTTTGGGTGACAACAACTCACTAGTTTGCCCATT

		TGGTGGATCTTGCGAAAATTTACAGCACGCTCTTCTATGGT TGCTCAATGCTCCCAATACGAAGTTAATAATTTCCCCAGTA TAATCCCTGCAAAAGATAAACACCTCACAATACACTTAATT GACTCGCCGTCCACCTCAGTACACTGTGCGATAGACAAGCT TATCGATACCGTCGACC
UQg58	mNeonGreen codon optimised	GATCTCCACAGAAAACTTCAAAATGGTCTCCAAGGGCGAGG AGGACAACATGGCCTCTCTCCCTGCCACTCACGAGTTGCAC ATCTTCGGCTCCATCAACGGTGTCGACTTCGACATGGTCGG TCAGGGCACCGGCAACCCTAACGACGGTTACGAGGAGTTGA ACCTTAAGTCCACCAAGGGTGACCTCCAGTTCTCCCCCTGG ATTCTTGTCCCTCACATCGGCTACGGCTTCCACCAGTACCT TCCCTACCCTGACGGCATGTCCCCTTTCCAGGCCGCCATGG TTGACGGCTCCGGTTACCAGGTCCACCGTACTATGCAGTTC GAGGACGGTGCCTCCCTTACTGTTAACTACCGTTACACCTA CGAGGGTTCCCACATCAAGGGTGAGGCCCAGGTCAAGGGCA CTGGTTTCCCTGCTGACGGTCCTGTCATGACCAACTCCCTT ACCGCTGCCGACTGGTGCAGGTCCAAGAAGACTTACCCCAA CGACAAGACCATCATCTCCACCTTCAAGTGGTCCTACACCA CTGGTAACGGCAAGCGTTACCGATCCACTGCCCGAACCACC TACACCTTCGCCAAGCCTATGGCCGCTAACTACCTTAAGAA CCAGCCCATGTACGTTTTCCGTAAGACCGAGCTCAAGCACT CCAAGACCGAGCTCAACTTCAAGGAGTGGCAGAAGGCCTTC ACCGACGTCATGGGCATGGACGAGCTTTACAAGTAAAAGGT TGAGCGCGTAATAGTGG
